# Fire up Biosensor Technology to Assess the Vitality of Trees after Wildfires

**DOI:** 10.3390/bios14080373

**Published:** 2024-07-31

**Authors:** Eleftherios Touloupakis, Isabela Calegari Moia, Raffaella Margherita Zampieri, Claudia Cocozza, Niccolò Frassinelli, Enrico Marchi, Cristiano Foderi, Tiziana Di Lorenzo, Negar Rezaie, Valerio Giorgio Muzzini, Maria Laura Traversi, Alessio Giovannelli

**Affiliations:** 1 Research Institute on Terrestrial Ecosystems, National Research Council, Via Madonna del Piano 10, 50019 Sesto Fiorentino, Italyalessio.giovannelli@cnr.it (A.G.); 2 Dipartimento di Scienze e Tecnologie Agrarie, Alimentari, Ambientali e Forestali—DAGRI, Università degli Studi di Firenze, Via San Bonaventura 13, 50145 Firenze, Italy; 3 Research Institute on Terrestrial Ecosystems, National Research Council, Research Area of Rome 1, Strada Provinciale 35d n. 9, Montelibretti, 00010 Rome, Italy

**Keywords:** biosensors, abiotic stress, wildfire, tree vitality

## Abstract

The development of tools to quickly identify the fate of damaged trees after a stress event such as a wildfire is of great importance. In this context, an innovative approach to assess irreversible physiological damage in trees could help to support the planning of management decisions for disturbed sites to restore biodiversity, protect the environment and understand the adaptations of ecosystem functionality. The vitality of trees can be estimated by several physiological indicators, such as cambium activity and the amount of starch and soluble sugars, while the accumulation of ethanol in the cambial cells and phloem is considered an alarm sign of cell death. However, their determination requires time-consuming laboratory protocols, making the approach impractical in the field. Biosensors hold considerable promise for substantially advancing this field. The general objective of this review is to define a system for quantifying the plant vitality in forest areas exposed to fire. This review describes recent electrochemical biosensors that can detect plant molecules, focusing on biosensors for glucose, fructose, and ethanol as indicators of tree vitality.

## 1. Introduction

Plants are exposed to various stress factors throughout their growth and development, from germination to the end of their life cycle. The ability to cope with them by modulating physiological mechanisms determines the ability to survive and maintain an ecological niche within an ecosystem under environmental constraints (i.e., resilience). The ability of plants to cope with stress is also exploited in nature-based solutions for climate change mitigation in urban areas, where plants have a pivotal role in human wellness and environmental safety [1]. Plant stressors are generally divided into two categories: abiotic and biotic stress. Abiotic stress refers to the adverse effects of non-living components on plant physiology caused by suboptimal or extreme environmental conditions such as heat, excess salt, flooding, water limitation, pollution, etc. The constant interaction between plants and environmental constraints disrupts the natural biological processes and eventually leads to increased susceptibility to disease, slowed development, or even death of the plant [2]. Trees control their functionality through acclimation mechanisms under suboptimal conditions by modulating photosynthesis, respiration, growth, nutrient transfer, and water. Over time, the acclimation response evolves into adaptive mechanisms to withstand extreme and persistent environmental stresses [3,4,5]. In this framework, there is an urgent need to explore the physiological mechanisms controlling plant acclimation and adaption strategies to develop new approaches and tools to improve ecosystem resilience and mitigation capacity, as recent forecasting models predict an increase in the frequency and severity of adverse environmental conditions.

Wildfires are one of the most important natural disturbances affecting plant ecosystems worldwide by impairing tree growth [6]. In Europe, fires are traditionally recurrent in southern countries characterized by a Mediterranean climate, and the EU Biodiversity Strategy for 2030 considers this threat as an “immediate priority” to be addressed. Recent evidence shows that 61% of wildfires affect forests, and around 25% of these areas are in the EU’s Natura 2000 biodiversity hotspots. Nevertheless, climate change is expected to further increase fire risk across Europe, as has already been the case over the last decade [7]. To tackle the problem of wildfires in these areas, the new EU Forest Strategy for 2030 strengthens measures to prevent forest fires and promote better resilience to climate change.

**Figure 1:**
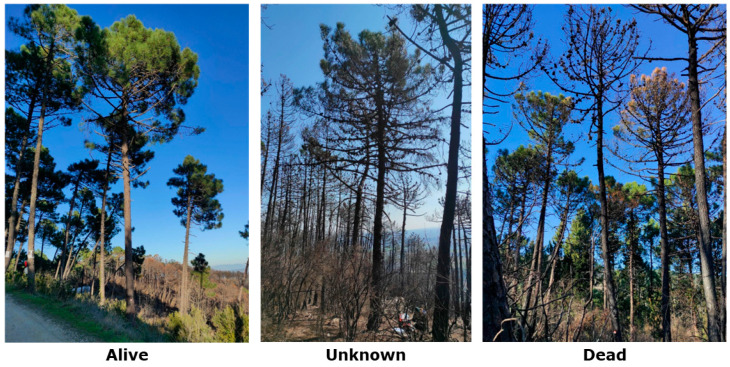
Three examples of Maritime pine plants located in the municipality of Vicopisano, Italy, damaged by fire. The plants were photographed in February 2022; the wildfire that damaged them was in August 2021. Trees that are considered alive and have green, unburnt foliage (**left**); trees with unknown vitality; they have both burnt and unburnt foliage (**center**); trees that are considered dead because they are completely burnt (**right**).

The damage that a fire causes to vegetation depends on the heat flows that are transferred to the different parts of the plant and is a function of several factors, such as fireline intensity, dwell time, and the rate of spread [8,9] (Figure 1). Within the same fire, fire behavior changes over time and space depending on the initiating factors (e.g., topography, meteorological conditions, fuel load, suppression measures, etc.), resulting in different heat fluxes and effects on the trees. High-intensity crown fires consume live and dead fuels, and burning of all the foliage and meristems in a tree crown can result in immediate tree death unless the tree can resprout from heat-resistant organs like epicormic sprouts (suckers) and adventitious shoots [10,11]. In contrast, low to moderate-intensity fires often do not pose a direct lethal threat to mature trees but can result in a variety of injuries that can affect their health. Cell death, caused by protein denaturation, is generally considered to be complete at 60 °C [12]. The rate of cell necrosis increases exponentially with temperature, and even lower temperatures can lead to cell death with prolonged exposure [13]. According to Bär et al. (2019), the tree may die immediately after the forest fire, or it may show signs of metabolic imbalance or progressive physical or biotic damage that may not become apparent until years later [14]. However, the response of plant functions to fire damage can vary greatly, also depending on the species, period of the year, tree age, etc. This means that trees that have survived the fire may exhibit different levels of physiological functionality, which may result in reduced growth or be more likely to succumb to delayed death [15,16].

**Figure 2:**
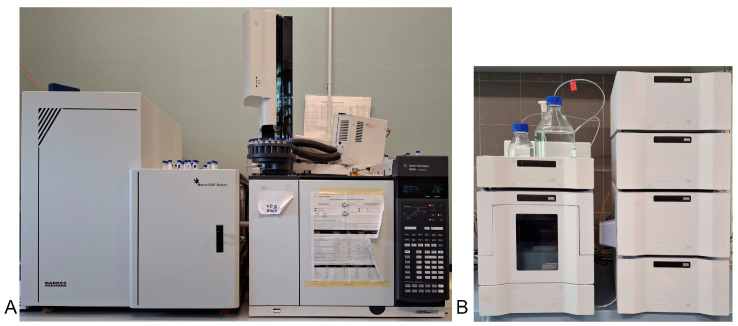
(**A**) The GC-MS (Agilent, Santa Clara, CA, USA) and (**B**) the HPLC (PerkinElmer, Waltham, MA, USA) instruments used at the CNR (Florence, Italy) for sugar analysis in trees.

**Figure 3:**
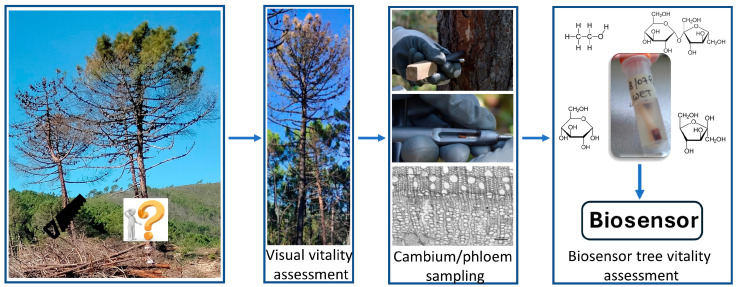
Schematic approach for the use of biosensors for the analysis of target molecules to assess the vitality of fire-damaged trees. The arrows indicate the sequential steps for determining the vitality of a tree damaged by a forest fire.

Currently, tree damage is assessed using empirical methods (visual or sensory assessment), anatomical observation of cambium vitality, or biochemical methods, which are not easy to apply in the field [14]. Nowadays, it is postulated that a few compounds, such as non-structural carbohydrates (soluble sugars and starch) or ethanol, can be considered valuable proxies for predicting the recovery of an injured tree after a wildfire [17,18]. Traditional biochemical monitoring methods for these compounds, such as gas chromatography-mass spectrometry (GC-MS) and high-performance liquid chromatography (HPLC), are sensitive and effective but time-consuming and require sample pretreatment and trained personnel (Figure 2). Practical monitoring programs require fast, simple, and cost-effective screening methods for the detection of proxies of tree health. Biosensors offer all these advantages as they can be easily deployed both in the laboratory and in the field (Figure 3).

The development of user-friendly new methods for assessing the vitality of fire-damaged trees should be very useful to support forestry professionals in planning and designing post-fire reforestation. In this context, this review describes some examples of enzymatic amperometric biosensors that can determine the concentration of signaling molecules in the stem tissue of injured trees and have been selected as indicators (proxies) of tree vitality to directly distinguish between damaged trees destined to die and those that are recovering.

## 2. Biosensors

**Figure 4:**
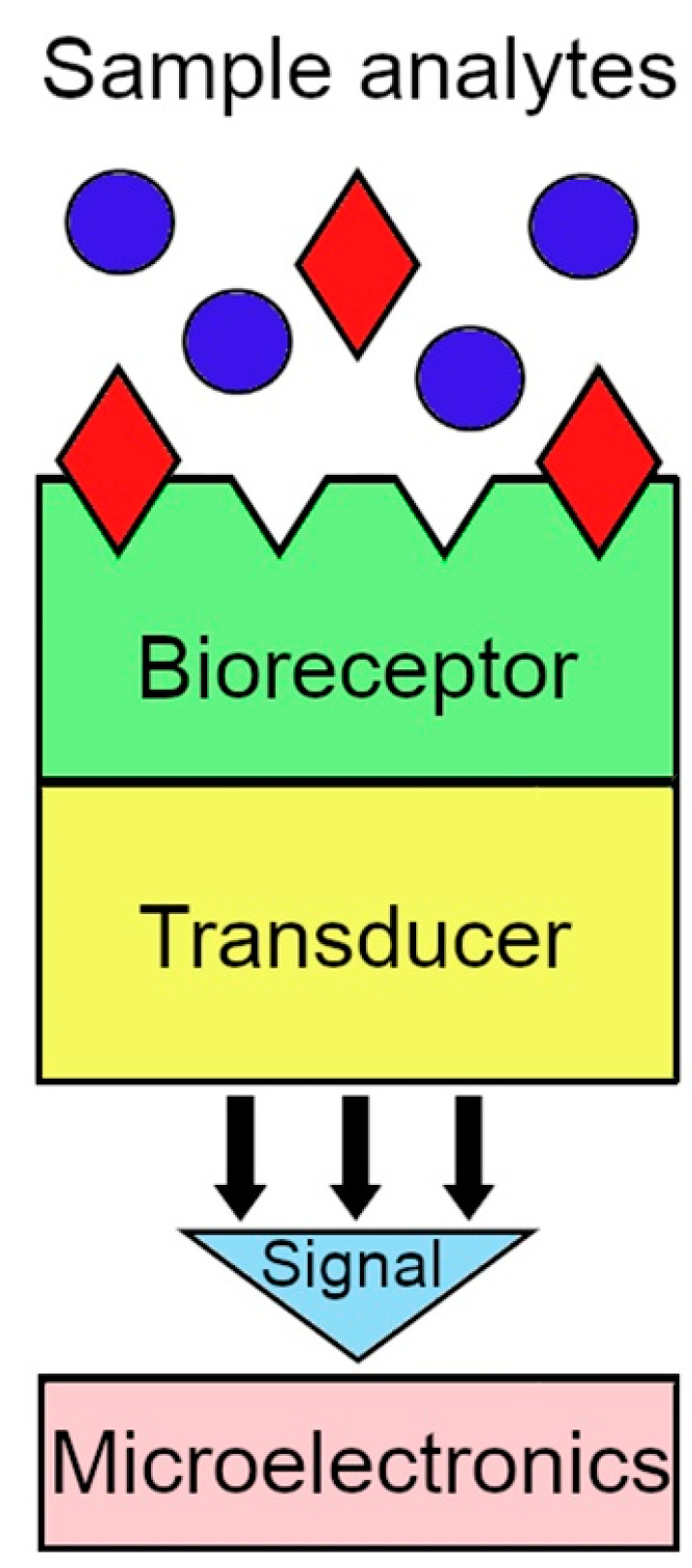
General scheme of a biosensor.

Biosensor technology offers fast, real-time testing and online measurements at low cost. The use of this technology as a forward-looking approach to understanding and controlling biological systems is extremely promising. A biosensor is an analytical instrument where a biological recognition element (bioreceptor) is coupled to a transducer (Figure 4).

The bioreceptor, also known as a biological recognition element, is generally an enzyme or a molecular receptor, such as an antibody, immobilized on the surface of the transducer. Biosensors can be categorized as immunochemical, enzymatic, non-enzymatic receptor, whole cell, or DNA biosensors based on the concept of biological recognition [19,20,21,22,23]. The role of the transducer is to convert the biological recognition event into a measurable signal that provides quantitative or semi-quantitative analytical information [24]. Specific interactions between the chemical target and the bioreceptor lead to a physico-chemical change that is recognized and measured by the transducer. In the presence of the analyte, the bioreceptor generates a signal that corresponds to its concentration. The ideal biosensor should be both selective and sensitive, have a low signal-to-noise ratio, provide a quantitative dose–response curve over physiologically relevant analyte concentrations, be non-invasive, and allow in vivo analysis [25].

The most used types of measurement are optical, electrochemical, and thermal [26,27,28,29,30,31]. The electronic circuit, the transducer, and the bioreceptor are the three basic components [32].

Biosensors have the potential to be used in a wide range of applications, such as clinical diagnosis by analyzing drugs and pharmaceuticals, mining and toxic gas monitoring, military and defensive applications, waste management by monitoring environmental pollution and microbial contamination, and personal safety in space travel [33,34,35]. Most commercially available biosensor systems are used in the pharmaceutical and healthcare industries [33,36,37,38]. A thorough understanding of biochemistry, electron flow, cofactor involvement, and interfering conditions is crucial for the development of an efficient system. A real-time safety monitoring system is ensured by the close connection between the detected signal, the triggered biological reaction, and the chemical contact. It is also important to separate the interaction of chemicals with bioreceptors from physical factors such as pH and temperature. One of the most important properties of the biosensor is the selectivity of the bioreceptor for the specific chemical target, which is maintained even in the presence of other potentially interfering species. The selectivity of biosensors, which enables real-time in-situ measurements, together with their small size, low cost, and wide range of potential applications, has attracted considerable commercial interest. Other advantages include high sensitivity, fast response time, minimal or no sample pretreatment, ease of use by non-experts, and the ability to regenerate and reuse the immobilized bioreceptor, enabling continuous or multiple testing. However, several problems associated with bioreceptors, such as their low stability and high cost, the need for cofactors, and difficulties with immobilization technology, have prevented their widespread commercialization.

## 3. Plant Biosensors

Monitoring the concentration of small molecules in plants is an important technique for obtaining early information on plant stress. Detecting dynamic changes in small molecule concentrations in situ is one of the most unique ways to obtain real-time plant health data. Plant health monitoring is a good strategy to support sustainable agriculture and increase crop yields while reducing the impact on the environment.

To date, novel biosensors have been proposed to monitor environmental conditions and plant growth, pesticide management, plant stress, and plant viruses [39,40,41,42,43,44,45]. Moreover, many electrochemical sensors have been developed for the in vivo detection of plant molecules [46,47,48,49].

Sneha et al. (2023) developed a biosensor based on an organic electrochemical transistor capable of detecting in vivo changes in ion concentration in plant sap [50]. The plant sap that flows through the xylem and phloem in the living plant was used as the electrolyte. In their recent study, Ruiz-Gonzalez et al. (2023) describe a sensor capable of simultaneously measuring K^+^ and plant sap pH in living plants using reverse iontophoresis and SPE modified by the deposition of a K^+^-selective membrane [51].

Plant-wearable sensors are increasingly being used in agriculture to monitor plant health. These devices can help detect diseases or abnormalities in plants at an early stage by monitoring their health in real time [52,53,54]. The ideal wearable sensor should be easily attached to different parts of the plant to monitor plant health on-site by collecting physiological data in real time. Wearable sensors for in vivo detection of small molecules in plants are either electrochemical sensors that extract molecules by reverse iontophoresis or implantable sensors that come into direct contact with the plant sap. The recorded data are converted into electrical signals and processed to monitor plant health by detecting abnormal changes in the concentration of small molecules.

Another interesting approach that has already been applied to the biosensing of plants is biosensing with microneedles [55]. Biosensors using microneedles can be non-invasive and portable, ensuring that the analysis takes place under normal plant development conditions. Microneedles can easily penetrate the plant epidermis and perform auxiliary work with high efficiency. They are less damaging to the plants, have a high monitoring sensitivity, and are easy to integrate [56]. Sensors with microneedles can detect the sap flow rate, collect the sap, and analyze its physicochemical parameters. For instance, Baek et al. developed a microneedle sap flow sensor to monitor water transport in tomato stems, and Jiao et al. (2019) developed a microneedle sensor to detect plant nitrate [57,58].

Recently, several wearable biosensors have been developed for the detection of glucose in plants [56]. They use the enzyme glucose oxidase (GO_X_), which catalyzes the oxidation of glucose to gluconolactone, which is then converted to gluconic acid. After electrochemical reduction to water or oxidation to oxygen, the oxygen in the solution is converted to hydrogen peroxide, which undergoes a redox reaction at the surface of the working electrode, resulting in a change in current. One of the main issues with this type of biosensor is the protection of the bioreceptors when the electrodes are inserted into the plant tissue. Researchers have used hydrogel-based microneedles as a new strategy to solve this problem [59,60]. Chen et al. (2024) recently presented an electrochemical microneedle sensor for continuous real-time monitoring of glucose in tomato and aloe vera plants. The sensor showed an LOD of 33.3 μM and a sensitivity of 17 nA/μM/cm^2^ [61]. Zheng et al. (2022) used hydrogel hollow silk fibroin-proline-based microneedles as a protective material for the bioreceptor [59]. During glucose detection, the shell of the hydrogel absorbs the solution and expands when it comes into contact with plant sap, allowing the liquid to penetrate the microneedles and come into contact with the electrode. The concentration ranges of glucose that the developed sensor was able to detect were 3–18 mM and 30–180 mM, with a sensitivity of 21.21 nA/mM. The sensor was able to determine the glucose content in a *Solanum lycopersicum* fruit without damaging it, with results similar to those of a blood glucose meter. Zhao et al. (2020) designed a microneedle-based biosensor for continuous glucose monitoring [62]. The device consisted of three pyramidal microneedles made of silk/D-sorbitol with immobilized GOx. The biosensor showed a linear range from 1.7 to 10.4 mM. The results suggest that microneedle biosensors are a promising technique for portable and continuous glucose monitoring.

Diacci et al. (2021) developed an implantable organic electrochemical transistor-based biosensor for the in vivo and real-time monitoring of glucose fluctuations in tree vascular tissue [63]. This is a proof-of-concept study in which implantable organic electrochemical transistor biosensors enable real-time monitoring of metabolites in plants and provide new insights into diurnal sugar homeostasis. The biosensor showed a linear range of 0.1 to 1.0 mM. This technology can be used to qualitatively analyze different metabolites and evaluate the effects of abiotic and biotic stress on them. In a recent work, Bukhamsin et al. (2022) presented a plant-wearable sensor with functionalized microneedle-based electrodes for the in situ detection of salicylic acid (LOD = 2.74 μM) [64]. They showed that their biosensor could serve as a promising platform for continuous and non-destructive monitoring in the field. Perdomo et al. (2023) developed a wearable electrochemical biosensor for the detection of glucose extracted from plant leaves through reverse iontophoresis [65]. This technology enables non-invasive in situ and in vivo identification of early stress responses in plants in real time and provides a unique tool for the timely agronomic management of crops. The biosensor showed a sensitivity of 22.7 nA/μM/cm^2^ and a limit of detection (LOD) of 9.4 μM. The same group recently presented another sensor system that enables non-invasive, real-time monitoring of salicylic acid levels in avocado trees [66]. The sensor with a reverse iontophoretic system and a graphene electrode showed a high sensitivity (82.3 nA/μM/cm^2^) and an LOD of 8.2 μM.

Dhanjai et al. (2020) developed a biosensor for the detection of gallic acid using a multilayer electrode prepared from layers of CNTs, cellulose nanocrystals, polyaniline, and 3-(glycidyloxypropyl)trimethoxysilane [67]. They showed that microneedles are promising for successful in situ screening of antioxidants in fruit matrices. Their biosensor showed a linear range from 0.58 to 512.6 μM with an LOD of 1.7 μM. Hossain and Tabassum (2022) developed a biosensor using a three-dimensionally printed, microneedle-based electrochemical sensor for real-time detection of salicylic acid [68]. Real-time analysis of salicylic acid in plant sap with an integrated pH correction function can help farmers respond promptly to environmental stress. The biosensor showed an LOD of 37 μM. Li et al. (2019) developed a biosensor containing a stainless-steel electrode fabricated with Au nanostructures, Pt nanoparticles, and reduced graphene oxide nanocomposite films, and a polymerized Safranine T film for in vivo detection of the phytohormone indole-3-acetic acid [69]. In vivo detection of plant signaling molecules such as indole-3-acetic acid is of great importance for precision farming, crop management, and plant phenotyping. The biosensor showed an LOD of 0.24 μΜ. Wang et al. (2021) developed a microneedle array biosensor based on Au@SnO_2_-vertical graphene (VG)/Ta for the detection of abscisic acid [70]. The small size, wide pH range, low LOD (0.004 μM), and wide linear concentration range (from 0.012 to 495.2 μM) allow the sensor to be used for in situ detection of abscisic acid in plants. Shao et al. (2023) developed a wireless and portable electrochemical sensor for the detection of indole-3-acetic acid in plants using screen-printed electrodes (SPEs) modified with gold nanoparticles and three-dimensionally reduced graphene oxide [71]. The sensor presented linear ranges from 0.25 to 120.0 μM and from 135 to 500 μM, and an LOD of 0.15 μM. They showed that the combination of modified SPE with small Bluetooth workstations and smartphones is very useful in creating a portable, low-cost, simple, and fast electrochemical sensing platform. Gao et al. (2021) fabricated an electrochemical sensor for the detection of free tryptophan by depositing a polydopamine-reduced graphene oxide-MnO_2_ nanocomposite onto a glassy carbon electrode [72]. They showed that their sensor could detect the tryptophan content in tomatoes in vitro and in vivo, demonstrating the feasibility of this research strategy for the development of electrochemical sensors for measurements in different plant tissues. The sensor presented a linear range of 1.0 to 300 μM and an LOD of 0.22 to 0.39 μM. Researchers have also developed a variety of enzyme-free glucose and fructose biosensors [73,74]. Table 1 shows some examples of electrochemical biosensors for the detection of plant molecules.

## 4. Sugars and Ethanol as Plant Signaling Molecules in the Stress Response

The production and distribution of sugars in different tissues at different stages of plant development are tightly regulated to meet the carbon and energy needs of the system, as sugars are necessary for development and metabolism and serve as both energy sources and structural components. They influence numerous genes involved in various metabolic processes, which has led to research focusing on the identification of sugar recognition and signal transduction pathways. Sugars are formed in mature leaves and then transported via the phloem to where they are needed or stored.

Sugars in plants, such as glucose and fructose, are important signaling molecules that regulate metabolic and physiological functions [75,76,77,78,79], and they also serve as messengers for hormones during the signal transduction process [80]. Changes in the environment can result in suboptimal conditions for plants, which in turn lead to changes in metabolic processes related to acclimation response. Impairment of photosynthesis is one of the most common responses of plants to reduced water availability, high temperatures, fire, or pollution [81,82], so fluctuations in soluble sugar content in the phloem, along the axial transport system of the stem and around the sinks can be expected. Fire-induced crown scorch and bud dieback, for example, lead to a decrease in CO_2_ fixation, impair sugar metabolism, and lead to a depletion of non-structural carbon (soluble sugars, starch, and lipids) in the reserve compartments of the tree. If the imbalance of sugar turnover in the phloem and ray parenchyma persists for a longer period of time, the recovery of the tree may be compromised [18]. Thus, maintaining a sufficient flux of soluble sugars in the phloem plays a crucial role in maintaining the vitality of a tree after a wildfire. The lethal damage caused by crown injury and disruption of phloem flux can, therefore, be detected by changes in the amount of sugar in the phloem, ray parenchyma, and sap composition (e.g., decrease in sucrose and increase in glucose and fructose due to caramelization).

The analysis of carbohydrate content in different tissues has been used to evaluate the vitality of trees [83]. The presence of starch and glucose in the tree organs can reflect the ability of a tree to withstand severe situations [84].

In the case of fire, ethanol production is related to many physiological mechanisms, such as respiration (O_2_ supply), membrane destabilization (reduction of aerobic respiration and fermentation enzymes), overall enzymatic activities, sap flow (poor O_2_ water and accumulation), which are highly temperature dependent and lead to toxic results for the cells [17]. Therefore, quantitative analysis of these components in plants is crucial. With advances in precision agriculture, the challenge is to develop technologies for in situ and on-site detection of sugars and alcohol in plants, as researchers often need to directly detect sugar content in plants [85]. Unfortunately, existing methods for determining sugar content in plants require tissue sampling, specialized manpower, and extremely complicated and expensive equipment and procedures [18,86,87]. In addition, they often require complex pretreatment of samples and a combination of different equipment and techniques. For example, to determine the content of non-structural carbohydrates and starch, 40 mg of dried powder (from the cambial region and the mature xylem) is extracted three times with 80% ethanol solution. For each extraction, the homogenates are gently vortexed and centrifuged. The supernatant and the resulting pellet are used to determine the content of non-structural carbohydrates (HPLC) or starch (spectrophotometric method) [87]. Martinez-Trinidad et al. (2010) compared various techniques for measuring tree vitality of live oaks [88]. The results of their study suggest that a portable blood glucose meter can accurately measure glucose levels. Given the relationship between glucose and starch levels, glucose content could be used to estimate the carbohydrate content of urban trees.

Plant signaling molecules such as glucose, fructose and ethanol in plants can be determined with electrochemical biosensors as indicators of tree vitality. The following sections are dedicated to the description of recently developed biosensors that can detect glucose, fructose, and ethanol.

### 4.1. Amperometric Glucose Biosensors

The glucose biosensor is one of the most important bioassay devices and has already been successfully commercialized [89]. Research to develop amperometric biosensors for glucose detection is enormous as they are relatively affordable, can be easily miniaturized, and require simple electronics. Kamanina et al. (2019) developed a glucose biosensor using modified SPEs with GOx and conductive hydrogel based on a sol-gel matrix and single-walled carbon nanotubes [90]. They have shown that high-performance biosensors can be developed using enzyme-modified SPEs and conductive hydrogel. The concentration range of glucose that the developed sensor could detect was 0.045–1.04 mM. Hu et al. (2022) developed a low-cost, simple-to-manufacture, and portable electrochemical glucose biosensor with modified SPEs [91]. The GOx enzyme was immobilized in graphene aerogel and Prussian blue-modified SPEs with chitosan. The combination of graphene aerogel and Prussian blue showed good conductivity and catalytic performance. The biosensor showed a linear range of 0.5–6.0 mM with an LOD of 0.15 mM. Sakalauskiene et al. (2023) developed a reagentless amperometric glucose biosensor by combining the graphite electrode modified with gold nanostructures and Prussian blue with GOx [92]. The biosensor was easy to use and had good repeatability. The LOD (8.8 μΜ) and linear range (from 0.025 to 1.0 mΜ) were suitable for glucose determination and showed high resistance to other electroactive substances present in the real samples. Liu et al. (2023) developed a non-invasive salivary glucose sensor consisting of a Nafion-carbon nanotube nanocomposite and GOx sequentially deposited on SPEs [93]. The developed sensor showed excellent selectivity in interference tests and good performance in the artificial saliva test. Their results showed a sensitivity of 99.13 μA/mM/cm^2^, a linear range of 20–700 μM, and an LOD of 20 μM. Liu et al. (2023) developed an amperometric glucose biosensor on a toothbrush [94]. They coated the toothbrush with carbon-graphite ink and Ag/AgCl ink as sensor electrodes, followed by immobilization of GOx. The biosensor detected glucose in a concentration range from 0.18 mM to 5.22 mM. The biosensor is promising for non-invasive monitoring of glucose levels in the saliva of diabetic patients. Albanese et al. (2014) developed amperometric glucose biosensors by using two methods for the deposition of Prussian blue and various membranes for the immobilization of GOx [95]. The aim of their work was to develop a suitable, stable, and cost-effective glucose biosensor based on a Prussian blue-modified SPE for food analysis. The biosensors prepared using silica sol–gel immobilization showed a linear range of 0.005 to 1.0 mM and an LOD of 20 μM. Khosravi et al. (2023) developed a glucose biosensor that can be applied to a textile substrate by screen printing [96]. The biosensor showed high selectivity to glucose and excellent stability over 30 days of storage. In addition, the biosensor showed a linear response in the range of 20–1000 μM, a high sensitivity (18.41 μA/mM/cm^2^) and an LOD of 20 μΜ. Ang et al. (2015) developed a biosensor for the detection of glucose in fruits by immobilizing GOx on a chitosan membrane [97]. The developed biosensor showed good repeatability and reproducibility. The results of the storage stability test indicated that the immobilization process allowed the enzyme to be reused, resulting in operational stability. The wide linear detection range (0.01–15 mM) ensures good accuracy in the measurement of glucose content.

### 4.2. Amperometric Fructose Biosensors

Several amperometric biosensors using immobilized D-fructose dehydrogenase (FDH) for the determination of D-fructose have been reported [21,98,99,100,101]. Some of them were based on platinum electrodes [102], glassy carbon electrodes [103], or carbon paste electrodes [104]. Biscay et al. (2012) developed a fructose biosensor based on ferrocyanide-modified SPEs [105]. The biosensor showed a linear response in the range of 0.1–1.0 mM, good sensitivity (1.25 μA/mM), and an LOD of 0.05 mM. Fructose was determined in real samples with good accuracy. Suzuki et al. (2020) developed a fructose biosensor by immobilizing a variant of FDH on a porous gold microelectrode [106]. The biosensor showed an LOD of 2.0 mM and a sensitivity of 200 ± 20 μA/mM/cm^2^, which was only dependent on temperature. Therefore, the sensor-enabled rapid detection without calibration at constant temperature. Trivedi et al. (2009) developed a low-cost, portable, and disposable fructose biosensor using FDH [107]. The biosensor showed a linear response in the range from 3 to 13 mM and an LOD of 0.65 μΜ. Siepenkoetter et al. (2017) developed a biosensor based on FDH on nanoporous gold electrodes [108]. After a very fast response time (<5 s), the biosensor showed accurate readings (linear response in the range of 0.05–0.3 mM and an LOD of 1.2 μM) and a high specificity for d-fructose in the presence of interfering sugars. Antiochia and Gorton (2014) developed a fructose biosensor based on an osmium-polymer modified graphene SPEs [109]. They have successfully produced a simple and low-cost biosensor that uses osmium polymer as both a mediator and a support material. The biosensor showed an LOD of 0.8 μM, a linear range from 0.1 to 8.0 mM, and high sensitivity (2.15 μA/mM/cm^2^). Bollella et al. (2018) developed a sensitive membrane-less fructose biosensor based on FDH immobilized on a highly porous gold electrode modified with aryl thiol [110]. The biosensor responded rapidly, showed great stability (>90% of the signal remained after 90 days), high catalytic current density (920 μA/cm^2^), selectivity and sensitivity (175 ± 15 μA/mM/cm^2^) with the lowest detection limit (0.3 μM).

### 4.3. Amperometric Ethanol Biosensors

Alcohol biosensors commonly use alcohol dehydrogenase (ADH) and alcohol oxidase (AOX) to detect alcohol [111,112,113]. ADH and AOX catalyze processes that can be easily measured with commercially available electrochemical transducers. The most commonly used alcohol biosensors are the AOX biosensors. AOX oxidizes low molecular weight alcohols to aldehydes using molecular oxygen (O_2_) as an electron acceptor: RCH_2_OH + O_2_ → RCHO + H_2_O_2_. Due to the strong oxidizing properties of O_2_, AOX oxidizes alcohols irreversibly. The consumption of O_2_ or the formation of H_2_O_2_ can be measured electrochemically with amperometric electrodes by monitoring either the anodic or cathodic reaction caused by the oxidation or reduction of molecules on the surface of the working electrode.

Many amperometric biosensors that use immobilized ADH or AOX for the determination of ethanol have been reported [114,115,116,117]. Bilgi and Ayranci (2018) developed an amperometric ethanol biosensor based on ADH [111]. The biosensor exhibited the following analytical characterization parameters: linear range of 178.5 to 1000 μM, an LOD = 53.5 μM, and a sensitivity of 0.432 μA/mM. The biosensor provided good results when analyzing ethanol in a commercial alcoholic beverage. Zhang et al. (2021) proposed a screen-printed biosensor for ethanol analysis in fermentation based on the development of a well-defined nanocubic structure of a nanocomposite of gold nanoparticles and nickel hexacyanoferrate [112]. The biosensor showed excellent sensitivity with a high anti-interference capability to ensure accurate detection in a viscous and colored fermentation broth. This biosensor also showed good reproducibility and storage stability with repeated use over 30 days. Istrate et al. developed a sensitive ethanol biosensor based on ADH immobilized on the surface of a modified SPE with a nanocomposite material [113]. The biosensor showed good sensitivity (44.6 ± 0.07 µA/mM/cm^2^), low LOD (10 μM), good reproducibility, and stability of up to 6 weeks. Stasyuk et al. (2022) presented an amperometric biosensor based on AOX and peroxidase-like nanozymes for ethanol determination [116]. The developed biosensor showed a high sensitivity (260 μA/mM/cm^2^), a linear range from 5 to 100 µM, fast response, and low LOD (1.5 µM). They observed a high correlation between the ethanol content in real samples determined with the proposed biosensor and the colorimetric reference method. An amperometric bi-enzymatic ethanol biosensor based on AOX and horseradish peroxidase was presented by Hooda et al. (2018) [117]. The biosensor showed a fast (8 s) and linear response to ethanol in the range of 0.01–50 mM, an LOD of 0.1 nM, storage stability of 190 days, and a sensitivity of 155 µA/mM/cm^2^. Recently, the same group developed another ethanol biosensor in which AOX was immobilized on gold nanoparticles [118]. The biosensor showed a linear response from 0.01 mM to 42 mM, an LOD of 0.1 nM, and a storage stability of 180 days. Table 2 shows some examples of electrochemical biosensors for the detection of glucose, fructose, and ethanol.

## 5. Perspectives

The responses of plants to environmental constraints can vary greatly depending on the species, tree age, intensity, and frequency of the events. This means that trees that survive a fire may exhibit different physiological functions, resulting in reduced growth or, more likely, delayed death [15,119,120]. On the other hand, it is known that damaged trees can also benefit from reduced competition in the short and medium term [121,122,123]. Therefore, assessing fire injury to trees and irreversible physiological damage by identifying reliable proxies is a crucial step in planning the best practices to mitigate the consequences of fires and accelerate the regeneration processes of trees and/or restore biodiversity. For example, immediate detection of injury to trees could improve the knowledge of the compounds’ dynamics of post-fire tree mortality and forest recovery, and in this contest, biosensors are the best tool.

## 6. Conclusions

The aim of this review was to define a system to quantify plant vitality in forest areas exposed to fire. The review describes recent electrochemical biosensors that can determine plant molecules, focusing on the biosensing of glucose, fructose, and ethanol as indicators of tree vitality. Based on a comprehensive review of the current literature on biosensor technology, we conclude that electrochemical biosensors could be useful in quantifying the effects of forest fires on plant vitality.

## Figures and Tables

**Table 1:** Examples of electrochemical biosensors for the detection of plant molecules.

Analyte	Bioreceptor	Sensitivity	Linear Range	LOD	Reference
Glucose	GOx	22.7 nA/μM/cm^2^	0–80 μM	9.4 μM	[65]
Gallic Acid	CNT-CNC@PANI/microneedle	nd	0.58–512.6 μM	1.7 μM	[67]
Salicylic acid	CuMOF	nd	50–1000 μM	37.4 μM	[68]
Abscisic acid	Au@SnO2-vertical graphene (VG)/Ta microelectrodes	1.460 μA/μM	0.012–495.2 μM	0.004 μM	[70]
Indole-3-acetic acid	AuNPs-3DGR modified SPEs	0.527 μA/μM	0.25–120 μM	0.15 μM	[71]
Fructose	Co_3_O_4_ thin film	495 μA/mM/cm^2^	0.021–1.74 mM	1.7 μM	[73]
Tryptophan	PDA/RGO-MnO2/GCE	0.39–1.66 μA/μM	1–300 μM	0.22–0.39 μM	[72]
Glucose	COOH-GR–COOH-MWNT–AuNPs	nd	5–80 mM	0.537 mM	[74]
Fructose	COOH-GR–COOH-MWNT–AuNPs	nd	2–20 mM	1.63 mM	[74]
Arabinose	COOH-GR–COOH-MWNT–AuNPs	nd	2–50 mM	1.811 mM	[74]
Mannose	COOH-GR–COOH-MWNT–AuNPs	nd	5–60 mM	4.903 mM	[74]
Xylose	COOH-GR–COOH-MWNT–AuNPs	nd	2–40 mM	0.693 mM	[74]
Galactose	COOH-GR–COOH-MWNT–AuNPs	nd	5–40 mM	2.105 mM	[74]
Salicylic acid	MIPs	0.0312 μA/μM/mm^2^	0–20 μM	2.74 μM	[64]

**Table 2:** Examples of electrochemical biosensors for the detection of glucose, fructose, and ethanol.

Analyte	Bioreceptor	Sensitivity	Linear Range	LOD	Reference
Glucose	GOx	1480 nA/mM	0.045–1.04 mM	0.015 mM	[90]
Glucose	GOx	nd	0.5–6.0 mM	0.15 mM	[91]
Glucose	GOx	99.13 μA/mM/cm^2^	20–700 μM	20 μM	[93]
Glucose	GOx	0.0817 μA/mM/cm^2^	0.18–5.22 mM	5 μM	[94]
Glucose	GOx	nd	0.025–1.0 mM	8.8 μM	[92]
Glucose	GOx	18.41 μA/mM/cm^2^	20–1000 μM	20 μΜ	[96]
Fructose	FDH	1.25 μA/mM	0.1–1.0 mM	0.05 mM	[105]
Fructose	FDH	200 μA/mM/cm^2^	nd	2.0 mM	[106]
Fructose	FDH	0.62 nA/μM	3–13 mM	0.65 μΜ.	[107]
Fructose	FDH	3.7 μA/mM/cm^2^	0.05–0.3 mM	1.2 μM	[108]
Fructose	FDH	2.15 μA/mM/cm^2^	0.1–8.0 mM	0.8 μM	[109]
Fructose	FDH	175 μA/mM/cm^2^	0.05–5.0 mM	0.3 μM	[110]
Ethanol	AOX	260 μA/mM/cm^2^	5–100 µM	1.5 µM	[116]
Ethanol	AOX	155 µA/mM/cm^2^	0.01–50 mM	0.1 nM	[117]
Ethanol	AOX	nd	0.01–42 mM	0.1 nM	[118]

## Data Availability

The data that support the findings of this study are available from the corresponding author upon reasonable request.
